# New 3-dimensional implant application as an alternative to allograft in limb salvage surgery: a technical note on 10 cases

**DOI:** 10.1080/17453674.2020.1755543

**Published:** 2020-05-12

**Authors:** Jong Woong Park, Hyun Guy Kang, June Hyuk Kim, Han-Soo Kim

**Affiliations:** a Orthopaedic Oncology Clinic, National Cancer Center, Goyang;; b Division of Convergence Technology, National Cancer Center, Goyang;;; c Department of Orthopaedic Surgery, Seoul National University Hospital, Seoul, Korea

Recently, there have been attempts to reconstruct bone defects using 3-dimensional (3D)-printed implants (Imanishi and Choong 2015, Wong et al. 2015, Liang et al. 2017, Wei et al. 2017, Park et al. 2018a, 2018b, Angelini et al. 2019). A 3D-printed, titanium alloy implant with an appropriate pore structure is biocompatible and personalizable in terms of the surgical location and extent (Wu et al. 2013, Guyer et al. 2016, Lee et al. 2016, Mumith et al. 2017, Li et al. 2018, McGilvray et al. 2018, Park et al. 2020).

3D-printed titanium alloy implants are often used alone to fill bone defects, despite the lack of clinical evidence to support their use. The following major limitations of the 3D-printed titanium alloy implant have been identified: (1) mechanical safety of the 3D implant is not guaranteed, especially with regard to fatigue strength; (2) the maximum printable size is limited by the metal 3D-printer, and is usually an approximate length of 20 cm; and (3) 3D-printing using composite materials is technically difficult; accordingly, a single titanium alloy is often used for implant fabrication. For example, the titanium alloy Ti6Al4V is an ideal material in terms of reducing the stress shield effect and improving biocompatibility, but has weak wear-resistance. Thus, it is difficult to fabricate an implant (including a joint) using a single titanium alloy material.

To take advantage of 3D-printed titanium alloy implants and overcome the aforementioned disadvantages, the 3D-printed implant may be combined with conventional orthopedic surgical instruments, such as the intramedullary nail, artificial arthroplasty implant, and tumor prosthesis. In other words, 3D-printed titanium alloy implants provide biocompatibility and personalized size-matched filling for bone defects, while orthopedic instruments give mechanical strength and durable joint function. This technical note explores experiences with 3D-printed implants and a prosthesis composite (3DiPC) approach in various surgical contexts.

## Patients

10 patients who underwent surgeries combining 3D-printed titanium implants and conventional orthopedic surgical instruments (i.e., the 3DiPC procedure; 7 pelvic and 3 long bones) were studied. 7 surgeries were performed for oncological reasons, including osteosarcoma (n = 2), Ewing sarcoma (n = 2), undifferentiated pleomorphic sarcoma of the bone (n = 2), and bone metastasis from renal cell carcinoma (n = 1). 3 surgeries were revision cases and included massive bone defects due to previous oncological surgeries (n = 2) and a car accident (n = 1). The 3DiPC procedures combined 3D-printed implants and conventional total hip arthroplasty (THA) implants (n = 6), a modular tumor prosthesis (n = 2), and intramedullary nails (n = 2) (Tables 1 and 2). All patients were followed postoperatively using the standard schedule and strategy for conventional limb salvage surgery.

**Table 2.** Details of limb salvage surgery

Patient no.	Reason for 3DiP	Surgery time (min)				Combined conventional implant
				3D-printed implant		
		Resection	Reconstruction	(XЧYЧZ mm^3^)	Weight (g)	
1	No commercial implant	224	220	108Ч82Ч126	573	Modular tumor prosthesis: proximal femur and acetabular cup
2	No commercial implant	280	81	105Ч135Ч178	649	Conventional THA
3	No commercial implant	214	113	117Ч88Ч170	352	Conventional THA
4	No commercial implant	NA	30	47Ч52Ч55	21	Conventional THA
5	No commercial implant	174	102	106Ч77Ч185	452	Conventional THA
6	No commercial implant	NA	92	124Ч112Ч168	472	Conventional THA
7	No commercial implant	NA	104	98Ч98Ч129	192	Conventional THA
8	Saving adjacent joint	102	104	52Ч40Ч158	350	Retrograde intramedullary nail
9	Saving adjacent joint	98	81	35Ч34Ч165	25	Intramedullary nail
10	Saving adjacent joint	65	108	35Ч17Ч200	207	Modular tumor prosthesis: proximal humerus

Abbreviations: 3DiPC, 3D-printed implant and prosthesis composite; NA, not applicable; THA, total hip arthroplasty.

**Table 1.** Patient demographics

Patient no.	Age/sex	Location	Side	Diagnosis	Tumor presentation	Cause of surgery	Follow-up (months)	Oncologic status
1	52/F	Pelvis	R	UPS of the bone	Recurred	Oncologic	21	DOD
2	47/M	Pelvis	L	Chondrosarcoma	Primary	Oncologic	33	NED
3	52/M	Pelvis	R	Osteosarcoma	Primary	Oncologic	24	AWD
4	50/F	Pelvis	R	Breast cancer	NA (mechanical failure)	Nononcologic	21	NA
5	28/M	Pelvis	L	Ewing sarcoma	Primary	Oncologic	12	DOD
6	47/F	Pelvis	R	Chondrosarcoma	NA (mechanical failure)	Nononcologic	10	NA
7	47/F	Pelvis	L	Major trauma	NA (mechanical failure)	Nononcologic	10	NA
8	54/F	Femur	R	UPS of the bone	Primary	Oncologic	9	NED
9	68/F	Femur	L	Renal cell carcinoma	Primary	Oncologic	7	NED
10	20/M	Humerus	R	Osteosarcoma	Primary	Oncologic	16	NED

Abbreviations: AWD, alive with disease; DOD, dead of disease; NA, not applicable; NED, no evidence of disease;

UPS, undifferentiated pleomorphic sarcoma.

For all patients, except 1 patient who underwent surgery using a 3D-printed reinforcement cage, bone cutting was performed with a 3D-printed surgical guide. All patients requiring surgery for an oncological diagnosis had negative bone margins with preoperatively planned distances. The 3D-printed implants fit perfectly into the bone defects created by multiplanar bone cutting. Depending on the surgical location and scale, the mean bone tumor resection times were 88 (65–102) and 223 (174–280) minutes for long bones and the pelvis, respectively.

For patients who underwent pelvic reconstruction, independent gait without moderate-to-severe pain was achieved in 6 weeks. Notably, 2 patients who underwent chemotherapy after limb salvage surgery showed delayed rehabilitation. For 2 patients who underwent femoral reconstructive surgery, both cases utilized an intramedullary nail for stability and thus immediate weight-bearing activity was allowed. Finally, a patient who underwent humeral limb salvage surgery showed limited shoulder motion, but all functions below the elbow were preserved. There were no complications related to the 3DiPC surgery, including infection and mechanical failure in short- and mid-term follow-up (range, 7–33 months).

## Surgical technique (Table 2)

### Implant design and fabrication

The design process was coordinated for customized 3D-printed implants and surgical bone-cutting guides through close communication between orthopedic oncologists and engineers. Computerized tomography (CT) and magnetic resonance imaging (MRI) scans with a thin section thickness of 1–2 mm were used in the design process. All medical images were stored in the Digital Imaging and Communications in Medicine format. A graphical 3D model was created, and a mirror technique and virtual resection were performed using MIMICS (Interactive Medical Image Control System; Materialise; Leuven, Belgium). The implants had both lattice and solid structures in order to enhance bone ingrowth and to support mechanical strength. The dode-thin mesh structure was applied as a lattice structure using Magics 22 (Materialise; Leuven, Belgium). After 3D-printed implant fabrication, a polishing process was completed to prevent abrasion or adhesion to a major neurovascular bundle.

The cutting guide was fabricated using a PolyJet-type 3D printer (OBJET30 Prime, Stratasys, Eden Prairie, MN, USA) with MED610 (Stratasys, Eden Prairie, MN, USA), a biocompatible resin. MED610 is a rigid, almost colorless material and was approved as a United States Pharmacopeia Class VI plastic because of its cytotoxicity, genotoxicity, and delayed hypersensitivity. For the bone-cutting guide, the maximum build size was 294 × 192 × 148.6 mm^3^ with an accuracy of 0.1 mm and a minimum layer thickness of 16 microns. For implant fabrication, 3D printing was performed with medical-grade titanium (Ti6Al4V-ELI Per ASTM 136) using a powder-based electron beam melting (EBM) 3D printer (ARCAM A1, Arcam AB, Mölndal, Sweden). For the titanium metal implant, the maximum build size was 200 × 200 × 180 mm^3^ with an accuracy of 0.2 mm. The MEDYSSEY Company (Jecheon, Korea) fabricated the implant and surgical guide, and certified a custom-made implant from the Ministry of Food and Drug Safety.

The 3D-printed implant may be combined with conventional orthopedic surgical instruments, such as the intramedullary nail, artificial arthroplasty implant, and tumor prosthesis. Bone cement was used to assemble the 3D-printed implant and a conventional prosthesis to create a 3DiPC. This cementation procedure was identical to the protocol used for an allograft-prosthesis composite (APC) without expecting bone ingrowth between a structural allograft, or a recycled autograft and prosthesis. The interface between the host bone and the 3D-printed implant was saved from cementation so as not to interfere with bone ingrowth into the implant. Matched screw holes in the 3D-printed implant and the conventional prosthesis (e.g., a cup for total hip arthroplasty) helped to ensure stability.

### Pelvis

**Figure 1.**
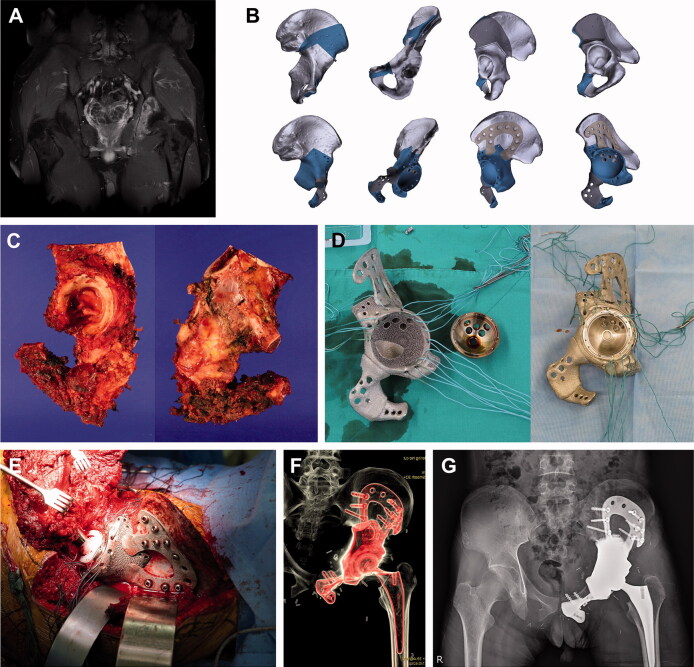
Pelvic reconstruction. Images of patient #5. (A) A preoperative gadolinium enhanced T1-weighted MR image showing Ewing sarcoma arising from the left acetabulum. (B) Graphical designs of the 3D-printed bone tumor resection guide (upper row) and implant (lower row). (C) Resected bone tumor as planned. Photographs of the (D) 3D-printed implant and THA cup before (left) and after conjugation (right). (E) Intraoperative photograph, (F) postoperative CT reconstruction image, and (G) plain radiograph showing pelvic reconstruction.

For pelvic reconstruction, conventional THA was performed using a 3D-printed pelvic implant generated via a metal-on-metal cementation technique. In other words, the 3D-printed metal implant provided an acetabular socket with a proper inclination and anteversion, and the 3D-printed implant and the THA cup were assembled using bone cement and screws through matched holes in both implants. The 3D-printed pelvic implants were individually fabricated to fit each patient and varied from a simple reinforcement cage to a megaprosthesis. The 3D-printed implant was mainly fixed by screws on the plates, which were fabricated integrally with the implant. The implant surfaces in contact with the host bone had a uniform lattice structure to enhance bone ingrowth. In most cases, bone-cutting guides were utilized to achieve a safe bone margin from the tumor and to fit the implant to the bone defect. The mean surgery time for pelvic reconstruction with the hip joint was 106 (30–220) minutes (Table 2; Figure 1).

### Femur diaphysis

**Figure 2.**
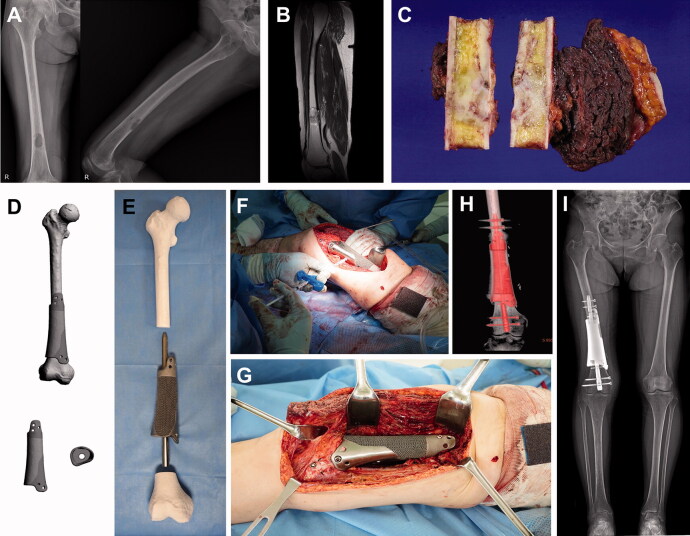
Femoral reconstruction. Images of patient #8. (A) Preoperative plain radiographs and (B) a T2-weighted MR image showing undifferentiated pleomorphic sarcoma of the bone arising from the distal femoral shaft. (C) Resected bone tumor as planned. (D) Graphical designs of the 3D-printed implant and (E) photograph of the 3D-printed implant and intramedullary nail to be used. (F) Intraoperative photograph showing cement injection through a premade hole. (G) Intraoperative photograph, (H) postoperative CT reconstruction image, and (I) teleradiogram showing femoral reconstruction.

For femoral reconstruction, an intramedullary nail was utilized to provide mechanical strength. The main role of the 3D-printed implant was bone-to-implant integration. To penetrate the 3D implant by an intramedullary nail, the implant had a tunnel inside, mimicking the bone marrow space. The tunnel for the intramedullary nail needed to match the nail contour reflected the bowing of the femur and had a diameter slightly larger than that of the nail. For the 1st patient to undergo femoral surgery (patient #8), the implant had a solid core structure coated with a lattice structure. The implant had a cuff circumference with a 1-cm depth for host bone insertion and a short plate with screw holes for additional rotational stability between the implant and host bone. To fill the gap between the 3D implant and the retrograde femoral nail, small side holes were made for bone cement injection. Although the retrograde femoral nail had a relatively straight shape, the reconstruction of a segmental defect with a single block of the 3D implant and a penetrating intramedullary nail was difficult (Figure 2).

**Figure 3.**
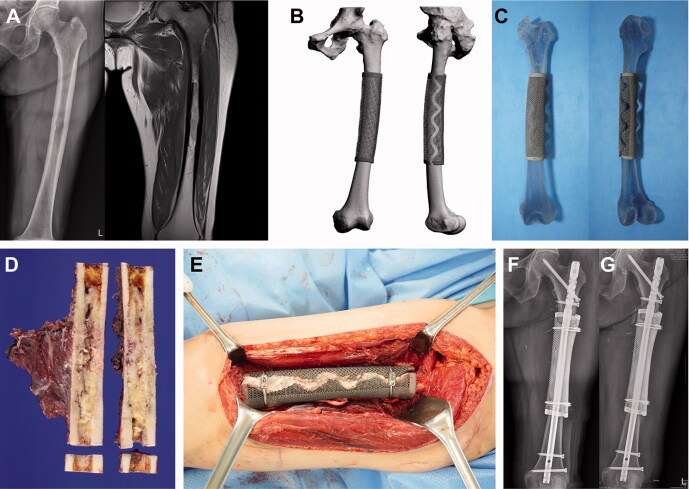
Femoral reconstruction. Images of patient #9. (A) Preoperative plain radiographs and a T2-weighted MR image showing metastatic renal cell carcinoma arising from the femoral shaft. (B) Graphical designs and (C) photograph of the 3D-printed implant. (D) Resected bone tumor as planned. (E) Intraoperative photograph and (F) postoperative plain radiograph showing femoral reconstruction. (G) Follow-up plain radiograph at 3 months postoperatively showing callus formation at both proximal and distal junctions.

Based on this experience, for the 2nd patient with femoral metastasis (patient #9), the 3D-printed implants to be used with intramedullary nails were generated with a full lattice (rather than solid) structured body. This implant reconstructed the cortical bone only and provided scaffolding for bone ingrowth, and mechanical stability was achieved by an intramedullary nail and bone cement. The cortical implant was made in 2 pieces and wrapped around the host bone junction in a telescopic manner. This approach to implant utilization enabled a simple intraoperative change in the cutting length according to the bone margin status within a few centimeters (Figure 3).

### Humerus

**Figure 4.**
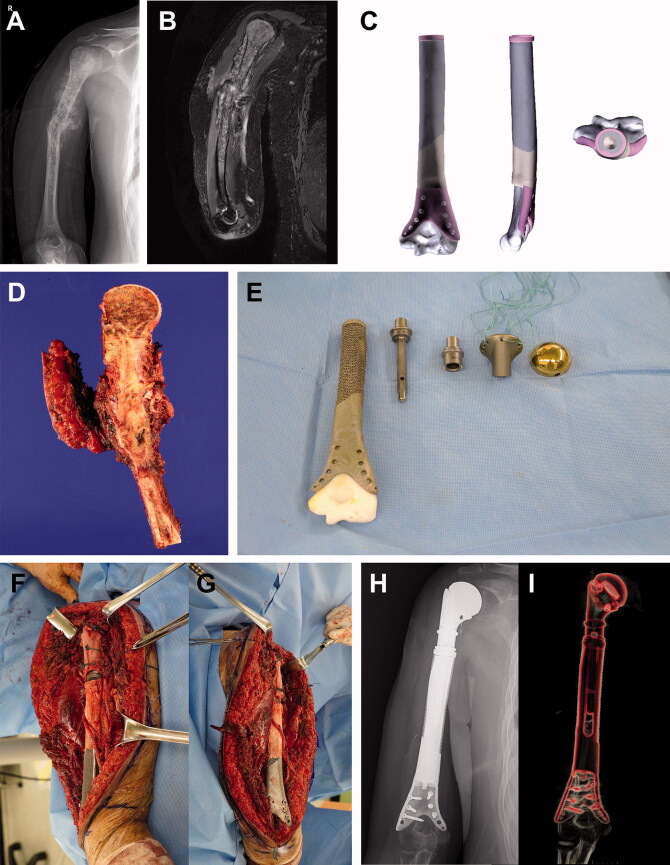
Humeral reconstruction. Images of patient #10. (A) A preoperative plain radiograph and (B) a T2-weighted MR image showing osteosarcoma in most of the humerus, except the elbow joint. (C) Graphical designs of the 3D-printed implant. (D) Resected bone tumor as planned. (E) Photograph of the 3D-printed implant and tumor prosthesis to be used. Intraoperative photograph showing (F) proximal and (G) distal parts of the reconstruction. (H) A postoperative plain radiograph and (I) CT reconstruction image showing humeral reconstruction.

Patient #10 had osteosarcoma in most of the humerus, except for a short segment above the elbow joint, and underwent limb salvage surgery using 3DiPC. This approach comprised a conventional proximal tumor prosthesis and a distal 3D-printed implant to preserve the elbow joint. Since the humerus is a non-weight-bearing bone and allows for shortening if necessary, the use of a 3D implant in combination with a conventional modular tumor prosthesis was a better surgical option than an intramedullary nail (Figure 4).

## Discussion

The 3D-printed titanium alloy implant is a personalized and biocompatible surgical option for massive bone defect reconstruction. However, this new type of implant still raises some concerns that need to be addressed. 1st, the long-term mechanical strength of an implant fabricated by 3D printing is not guaranteed. 2nd, a material containing a single titanium alloy theoretically would not have durable wear resistance for joint reconstruction, and 3D-printing using 2 or more materials is technically difficult at this time. 3rd, there is a printing size limitation (∼20 cm length). Therefore, it is necessary to use conventional internal fixation devices, arthroplasty implants, or modular tumor prostheses with plenty of clinical experience to supplement 3D-printed implants during limb salvage surgery.

Historically, APC has been used to reconstruct large bone defects (Mankin et al. 1996, Wunder et al. 2001, Jeon et al. 2007, 2014). Although bone stock restoration is one of the most important advantages to using APC, the effect may not be significant (Wilke et al. 2019). The 3D-printed titanium alloy implants with appropriate internal pores not only have a bone conduction effect, but also avoid problems related to osteolysis; thus, these implants may be mechanically stronger and longer lasting than structural allografts. Therefore, 3D-printed implants with adequate internal pore structures could be considerable alternatives to allografts using the APC technique.

The weight of 3D-printed implants varies depending on the bone defect size and mechanical strength requirements. In the pelvic area, a large bone defect including the acetabulum required a large-sized implant with great mechanical strength; thus, the mean weight was 500 (352–649) g. However, in the pelvic area, even a relatively small-sized reinforcement cage-type implant weighed between 21 and 192 g. In the case requiring 3DiPC with an intramedullary nail, the mechanical strength was reinforced by an intramedullary nail before bone incorporation into the 3D-printed implant, and the 3D-printed implant mainly acted as a scaffold for the fusion of the bone and soft tissue. Therefore, for the last patient who underwent limb salvage surgery for the distal femur, the 3D-printed implant consisted of a full-mesh body type with minimal mechanical strength and weighed only 25 g (Table 2).

**Figure 5.**
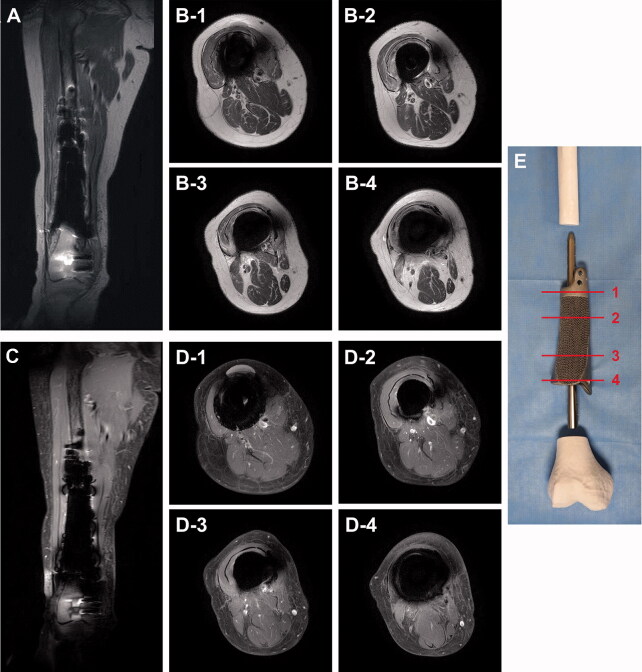
Metal artifacts in postoperative MR images. Metal artifacts around the titanium alloy (Ti6Al4V) megaprosthesis were not severe on postoperative MRI, and T2-weighted MR images (A, B) were clearer than enhanced T1-weighted MR images (C, D). Axial MR images were presented at the proximal solid cuff (B-1, D-1), proximal shaft with full lattice coating (B-2, D-2), distal shaft with anteromedial half-lattice coating (B-3, D-3), and distal solid cuff (B-4, D-4). (E) A photograph showing reference lines for axial images.

A lattice-structured body is a unique advantage of a product fabricated by 3D-printing technology. Although a lattice-structured body is mechanically weaker than a solid body, it provides a scaffold to enhance bone conduction and prevents the stress-shielding effect on the host bone. In addition, this structure also reduces metal-induced artifacts, thus enabling the magnetic resonance (MR) surveillance of postoperative tumor recurrences. Titanium is known to cause fewer artifacts on MR images relative to other metals due to its lower susceptibility (Hargreaves et al. 2011, Dillenseger et al. 2016). In previous literature, the metal artifacts caused by 3D-printed titanium alloy implants (Ti6Al4V) were not severe. To our knowledge, no previous studies have shown that the lattice structure reduces metal-induced artifacts in MR images. However, the reducing effect of the lattice structure has been observed. Specifically, a solid body coated with a few-millimeters-thick lattice structure yielded better quality postoperative MRI images of tissues around the implant than a pure solid structure without a lattice structure (Figure 5).

This technical note has some limitations. The small number of patients and single-institution design may limit the generalizability of the study results. The short follow-up period made it difficult to ascertain the local recurrence rate after wide excision and implant longevity. Long-term follow-ups involving clinical and biomechanical data subjected to dynamic finite element analyses and experimentation are needed to clarify the mechanical properties of 3D-printed implants. One major advantage of using 3DiPC rather than APC may be reduced surgical time due to omitting allograft carving and easy fixation by the preoperatively fabricated fixation part of the implant. However, proper comparison while controlling for confounding factors related to surgery time has not been done.

In conclusion, 3D-printed implants provide another surgical option involving the 3DiPC approach. This approach could resolve some concerns regarding the use of a new type of 3D-printed implant, such as possible mechanical weakness, lack of fatigue strength, weak wear resistance, and limitation of the maximal printable size.

### Ethics, funding, and potential conflicts of interest
